# Supplementary material for: COZOID: contact zone identifier for visual analysis of protein-protein interactions
Source: BMC Bioinformatics. 2018 Apr 6;19:125. doi: 10.1186/s12859-018-2113-6 (PMC5889581; doi:10.1186/s12859-018-2113-6)
Supplement: Supplementary file 2 — Software build. Executable binary file of the software tool. (ZIP 118,784 kb) [file 12859_2018_2113_MOESM2_ESM.zip › cozoid/platform/docs/testng-6.5.1-javadoc.zip!/org/testng/annotations/Optional.html]

Optional (TestNG)


---


|  |  |  |
| --- | --- | --- |
| **Overview** \| **Package** \| **Class** \| **Tree** \| **Deprecated** \| **Index** \| **Help** | |  |
| **PREV CLASS**   **NEXT CLASS** | **FRAMES**    **NO FRAMES**     **All Classes** |  |
| SUMMARY: REQUIRED \| OPTIONAL | DETAIL: ELEMENT |  |


---


## org.testng.annotations Annotation Type Optional

---

``` @Retention(value=RUNTIME) @Target(value=PARAMETER) public @interface Optional ```

Specifies that the current parameter is optional. TestNG will pass
in a specified default value, or `null` if none is specified.

---

| **Optional Element Summary** | |
| --- | --- |
| `java.lang.String` | `value`             The default value to pass to this parameter. |

### value

```
public abstract java.lang.String value
```

:   The default value to pass to this parameter.

    The default deserves
    a bit of explanation. JSR-175 (which defines annotations) says that
    Java annotation parameters can only be ConstantExpressions, which
    can be primitive/string literals, but not `null`.

    In this case, we use this string as a substitute
    for `null`; in practice, TestNG will pass `null`
    to your code, and not the string "null", if you do not specify
    a default value here in this parameter.

    :   **Default:**: "null"


---


|  |  |  |
| --- | --- | --- |
| **Overview** \| **Package** \| **Class** \| **Tree** \| **Deprecated** \| **Index** \| **Help** | |  |
| **PREV CLASS**   **NEXT CLASS** | **FRAMES**    **NO FRAMES**     **All Classes** |  |
| SUMMARY: REQUIRED \| OPTIONAL | DETAIL: ELEMENT |  |


---
